# Genetic Association of *ACE2* rs2285666 Polymorphism With COVID-19 Spatial Distribution in India

**DOI:** 10.3389/fgene.2020.564741

**Published:** 2020-09-25

**Authors:** Anshika Srivastava, Audditiya Bandopadhyay, Debashurti Das, Rudra Kumar Pandey, Vanya Singh, Nargis Khanam, Nikhil Srivastava, Prajjval Pratap Singh, Pavan Kumar Dubey, Abhishek Pathak, Pranav Gupta, Niraj Rai, Gazi Nurun Nahar Sultana, Gyaneshwer Chaubey

**Affiliations:** ^1^Cytogenetics Laboratory, Department of Zoology, Banaras Hindu University, Varanasi, India; ^2^Prosthodontics Unit, Faculty of Dental Sciences, Institute of Medical Sciences, Varanasi, India; ^3^Department of Neurology, Institute of Medical Sciences, Banaras Hindu University, Varanasi, India; ^4^Scangene Labs Private Limited, New Delhi, India; ^5^Birbal Sahni Institute of Palaeosciences, Lucknow, India; ^6^Centre for Advanced Research in Sciences (CARS), Genetic Engineering and Biotechnology Research Laboratory, University of Dhaka, Dhaka, Bangladesh

**Keywords:** *ACE2*, India, rs2285666, coronavirus, COVID-19, SARS-CoV-2

## Abstract

Studies on host-pathogen interaction have identified human ACE2 as a host cell receptor responsible for mediating infection by coronavirus (COVID-19). Subsequent studies have shown striking difference of allele frequency among Europeans and Asians for a polymorphism rs2285666, present in *ACE2*. It has been revealed that the alternate allele (TT-plus strand or AA-minus strand) of rs2285666 elevate the expression level of this gene upto 50%, hence may play a significant role in SARS-CoV-2 susceptibility. Therefore, we have first looked the phylogenetic structure of rs2285666 derived haplotypes in worldwide populations and compared the spatial frequency of this particular allele with respect to the COVID-19 infection as well as case-fatality rate in India. For the first time, we ascertained a significant positive correlation for alternate allele (T or A) of rs2285666, with the lower infection as well as case-fatality rate among Indian populations. We trust that this information will be useful to understand the role of *ACE2* in COVID-19 susceptibility.

## Introduction

The progressive and rapid spread of the novel coronavirus SARS-CoV-2, has created a worldwide wave of crisis by profoundly affecting the human health, and global economic stability. Recent researches have shown that the ACE2 (encoding Angiotensin-Converting Enzyme 2) is the main host cell receptor of spike glycoprotein responsible for the infection (Hoffmann et al., 2020; Lu et al., 2020; Zhou et al., 2020). It plays a crucial role for the entry of the virus into the cell to cause the final infection (Lu et al., 2020). ACE2 is a type I transmembrane metallocarboxypeptidase with homology to ACE, an enzyme long-known to be a key player in the Renin-Angiotensin system (RAS), and a target for the treatment of hypertension (Li et al., 2003; Shi et al., 2014). The secreted protein catalyzes the cleavage of the C-terminal dipeptide of Angiotensin I to produce Angiotensin 1–9 and Angiotensin II to Angiotensin 1–7.

The ACE2 is mainly expressed in vascular endothelial cells, the renal tubular epithelium, and in Leydig cells of the testes (Riordan, 2003; Kuba et al., 2010). PCR based analyses have revealed that ACE2 is also expressed in the lung, kidney, gastrointestinal tract, and blood vessels, tissues (Harmer et al., 2002; Ksiazek et al., 2003; Jiang et al., 2014). More recent reports have suggested ACE2 expression in the mucosa of the oral cavity may grant easy access to the virus for a new susceptible host (Xu et al., 2020). This explains the high incidence of pneumonia and bronchitis in those with a severe SARS-CoV-2 infection (Zhou et al., 2020). The ACE2 regulates blood volume, systemic vascular resistance, and thus cardiovascular homeostasis. *ACE2* has previously been found to be associated with hypertension, stroke, dyslipidemia, cardiovascular diseases, and kidney diseases (Wang et al., 2014; Pan et al., 2018; Wu et al., 2018; Zhang et al., 2018). People on ACE inhibitors and ARBs (angiotensin II type I receptor blockers) produce more numbers of receptors, raising the question of increased susceptibility to the infection, as the SARS-CoV-2 attaches itself to the ACE receptors on heart and lung tissues (Mehra et al., 2020). The expression of ACE2 is also substantially increased in patients with type 1 or type 2 diabetes, who are treated with ACE inhibitors and ARBs (Fang et al., 2020).

Contemporary studies on the large number of samples have analyzed the genomic variations present among populations worldwide (Cao et al., 2020; Stawiski et al., 2020). It was unanimously shown that a polymorphism rs2285666 present in *ACE2*, varied significantly among European and Asians (Asselta et al., 2020; Cao et al., 2020). The expression experiments have suggested that the alternate allele (TT-plus strand or AA-minus strand) of rs2285666 elevated the expression of this gene upto 50%, hence may play a role in SARS-CoV-2 susceptibility (Wu et al., 2017; Asselta et al., 2020). Therefore, we studied the haplotype structure as well as association of this allele for COVID-19 susceptibility in India. In particular, we have analyzed the haplotypes downstream to rs2285666 among worldwide populations as well as compared the frequencies of this allele and number of cases and case-fatality rate in India for any existing association.

## Materials and Methods

The worldwide data for haplotype analysis was extracted from recent complete genome analysis (Pagani et al., 2016). NETWORK 5 (Bandelt et al., 1999) was used to construct the median joining network of haplotypes derived from rs2285666 polymorphism. All the SNV’s observed downstream to rs2285666 have been listed in Supplementary Table 1. Illumina HumanHap 730K genotype chip has listed rs2285666 SNV in their panel, therefore we looked the genotype data generated on this platform. The frequency data of SNV rs2285666, of various populations were extracted by using Plink 1.9 (Chang et al., 2015), from 1,000 genome project data phase 3 (1000 Genomes Project Consortiumet al., 2010), data published by Estonian Biocentre (Chaubey et al., 2017; Pathak et al., 2018; Tätte et al., 2019; Estonian Biocentre Public _Data, 2020) and our newly genotyped samples for various Indian states and the Bangladesh (Supplementary Table 2). The frequency maps were generated by https://www.datawrapper.de/. The regression plots were constructed by https://www.graphpad.com/quickcalcs/linear1/ and verified by the Microsoft excel regression calculations. We have also used Pearson’s correlation coefficient test (Benesty et al., 2009) to validate our results. SPSS (ver 25) was used to estimate the Person correlation for two tailed significance test at 95% CI and 1,000 bootstrapping (2,000,000 seeds). The joint plots for all parameters were obtained from customized script of program R (ver 4) (R Core Team, 2012).

## Results and Discussion

Studies have shown that angiotensin-converting enzyme 2 (ACE2) acts as an entry receptor for coronavirus (Li et al., 2003). The interaction between SARS-S and ACE2 has already been expounded at molecular level in detail suggesting ACE2 as key determinant of SARS-CoV transmission (Li F. et al., 2005; Li W. et al., 2005). It has been revealed that human recombinant soluble ACE2 (hrsACE2) inhibits growth of SARS-CoV-2 and interrupts early stages of infections (Monteil et al., 2020). The variable susceptibility to the SARS-CoV-2 infection may be associated with the certain genomic variants within *ACE2*, that modulate its function or expression.

Among all the common exonic variants, some very recent studies done on *ACE2* variants reported population-based frequency differences for a single nucleotide variant (SNV) rs2285666 (also called G8790A) (Asselta et al., 2020; Cao et al., 2020; Strafella et al., 2020). This variant of *ACE2* was significantly different for Europeans (0.2), than the East Asians (0.55) (Asselta et al., 2020; Cao et al., 2020). Our analysis on Indian population has revealed mean frequency ∼0.6 of this allele (Srivastava et al., 2020). Moreover, our haplotype analysis for this gene revealed excessive sharing of the frequent South Asian haplotypes with East Eurasian populations, rather than West Eurasian populations (Srivastava et al., 2020). Similar to the East Asians, we have also noted that the frequency of this allele is significantly higher (two tailed *p* < 0.0001) among Indian populations in comparison with either of European, American, or African.

Since rs2285666 has already been proven to be a potential risk factor for hypertension, type 2 diabetes, and coronary artery disease (Chaoxin et al., 2013; Asselta et al., 2020), therefore, may possibly be a predisposing factor associated with the comorbidities observed in COVID-19 patients. Variant rs2285666 is located at the beginning of the intron 3, theoretically affecting gene expression with alternative splicing mechanisms (Li, 2012; Yang et al., 2015). A study also reported for the association of three rs2285666 genotypes with ACE2 protein level measured in serum by ELISA, with the A/A genotype having an expression level almost 50% higher than the G/G genotype (Li, 2012). More recently, it is shown that the substitution of G with A is predicted to increase the strength of the splice site of about 9.2%, resulting higher expression of ACE2 protein (Asselta et al., 2020). It has been also noted that patients characterized by higher ACE1 activity (a protein similar to ACE2) in conjunction with reduced ACE2 activity (i.e., CC/GG females or hemizygous C/G-males for rs2285666) account for increased susceptibility to hypertension, mainly in association with classical cardiovascular risk factors such as old age, dyslipidemia, and diabetes (Pinheiro et al., 2019; Ghafouri-Fard et al., 2020). Thus, it is clear that decreased ACE2 level contributes to severe consequences of SARS-CoV-2 infection (Samavati and Uhal, 2020; Verdecchia et al., 2020).

**FIGURE 1:**
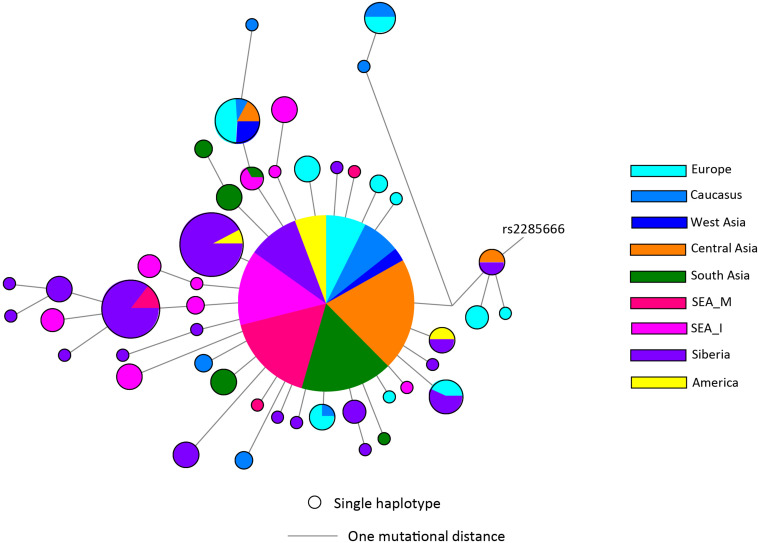
The median joining (MJ) network analysis constructed from haplotypes downstream to variant rs2285666. The network was constructed manually from the analysis obtained from NETWORK 5 pogramme. SEA_M, Southeast Asian Mainland; SEA_I, Southeast Asian Island. The downstream polymorphisms of CNV rs2285666 have been shown in Supplementary Table 1.

**FIGURE 2:**
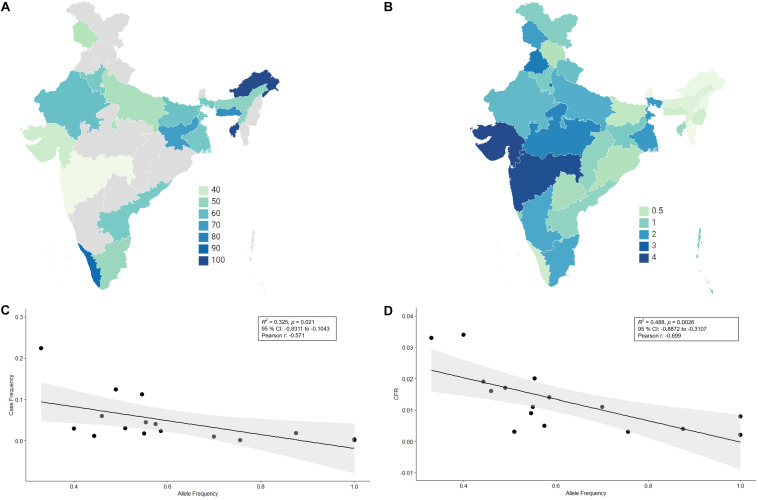
**(A)** The frequency (%) of allele rs2258666 among Indian populations. The gray colored areas in the map show the absence of data. **(B)** The statewise frequency (%) of case-fatality rate (CFR) (updated till 23rd August 2020). **(C)** The regression analyses showing the goodness of fit and Pearson correlation coefficient for the allele frequency and frequency of cases as well as **(D)** allele frequency vs. CFR (case-fatality rate) among various states of India (updated till 23rd August 2020).

There has not been any study so far on this SNV among Indian Populations. Therefore, we first looked the haplotype sharing, derived after variant rs2285666 among worldwide populations (Figure 1). Most of the haplotypes downstream to variant rs2285666 were belonging to South Asian, Central Asian, and East Eurasian populations. The starlike structure of rs2285666 derived haplotype indicate a case of positive selection among Asian populations, which needs further exploration (Figure 1 and Supplementary Table 1). Subsequently, our spatial analysis showed that in India, frequency of alternate allele of this SNV (rs2285666) varied between 33% and 100% (Figure 2A and Supplementary Table 2). The frequency gradient (lower to higher) is observed from Northwestern and Western region to Northeastern part of the subcontinent. In order to understand the correlation of allelic frequency with respect to the frequency of cases among Indian populations (Figure 2B), we performed linear regression and Pearson’s correlation coefficient analyses for variant rs2285666 and frequency of cases as well as case-fatality rate (CFR) (Figures 2C,D and Table 1). The regression analysis showed a significant correlation between allele frequency and number of cases (*p* < 0.05) (Figure 2C and Table 1). More number of cases are observed where frequency of this allele is lower and vice versa. The goodness of fit (R^2^) explained 34.6% of the variation. This suggests that the effect size of this allele for Indian populations is large. Since this is an ongoing pandemic and the number of infected people changes with time, we tested this result by adding the latest number of cases (August 2020) as well as the CFR (Figure 2 and Table 1). The latest data is also consistent with the older observation. We didn’t find any significant difference between both of the results. Moreover, the CFR data showed stronger association with the allele frequency of rs2285666 (Table 1). Further, in order to confirm our findings, we have also performed the Pearson’s correlation coefficient test. The genetic variation (frequency of rs2285666) and number of cases are negatively correlated with *r* = −0.571, *p* = 0.05 (August 2020), as well as frequency of rs2285666 and CFR are also negatively correlated with *r* = −0.699, *p* = 0.005 (Figures 2C,D and Table 1). The Pearson correlation analyses supported previous observations by showing a strong negative correlation of rs2285666 (allele T/A) with the number of cases and case-fatality ratio.

**TABLE 1:** Outcome of the various tests performed for statistical significance.

**Observation**	**Linear regression**		**Pearson’s correlation**	
	**R square**	***p*-value**	**r**	***p*-value**
May 2020_Cases	0.346	0.0165	−0.588	0.0167
August 2020_Cases	0.325	0.021	−0.571	0.021
August 2020_CFR	0.488	0.002	−0.699	0.002

Thus, for the first time, we showed a strong correlation of alternate allele (allele T on plus strand or allele A on minus strand) of variant rs2285666 with the lower infection rate as well as lower CFR among Indian populations. Although whole genome sequencing of a considerably large sample of cases and control individuals in India need to be performed to secure a robust genetic information on susceptibility for the disease, we here establish a possibility of the SNV (rs2285666) being associated with a protective role against COVID-19.

We caution that this is just one of the factors affecting the transmission, however there are several other elements (e.g., variation of rs2285666 among diverse ethnic groups of a state, sex of a person, comorbidity, virus strain, temperature, humidity, population density, social distancing, lockdown, etc.), which can perturb the infection rate and CFR substantially. If more of genetic factors or polymorphisms are recognized that may have played a significant impact on the variability of SARS-CoV-2 course, it would be worthwhile to design a cheap and accurate DNA based test for coronavirus susceptibility.

## Data Availability Statement

All datasets generated for this study are included in the article/Supplementary Material.

## Author Contributions

GC concived and designed this study. AS, AB, DD, RP, VS, NK, NS, PS, PD, AP, PG, NR, and GS collected the data for allele and COVID-19. AS, AB, PS, PD, AP, and GC analyzed the data. AS, AB, DD, PS, and GC wrote the manuscript from the inputs of other co-authors. All authors contributed to the article and approved the submitted version.

## Conflict of Interest

PG was employed by the company Scangene Labs Private Limited. The remaining authors declare that the research was conducted in the absence of any commercial or financial relationships that could be construed as a potential conflict of interest.

## Supplementary Material

Supplementary Table 1 The details of SNV’s downstream to the rs2285666 polymorphism. This data is obtained from the Network analysis.

Supplementary Table 2 The summary of data used in present analyses.
