# Supplementary material for: Genetic Association of ACE2 rs2285666 Polymorphism With COVID-19 Spatial Distribution in India
Source: Front Genet. 2020 Sep 25;11:564741. doi: 10.3389/fgene.2020.564741 (PMC7545580; doi:10.3389/fgene.2020.564741)
Supplement: Supplementary Table 1 — The details of SNV’s downstream to the rs2285666 polymorphism. This data is obtained from the Network analysis. [file Data_Sheet_1.PDF]

S. No.	rs number	Build 37	Build 38	Ref Allele	Alternate Allele	Functional Consequence	Europe	Caucasus	West Asia	Central_Asia	South_Asia	SEA_M	SEA_I	Siberia	America
1	rs182366225	15579294	15561171	C	T	intron_variant, 3_prime_UTR_variant	0.000	0.000	0.000	0.000	0.000	0.000	0.044	0.000	0.000
2	rs233574	15582609	15564486	T	C	intron_variant	0.668	0.603	0.350	0.792	0.940	1.000	0.956	0.935	0.846
3	rs2074192	15582790	15564667	C	T	intron_variant	0.391	0.372	0.125	0.250	0.300	0.528	0.400	0.407	0.577
4	rs181390351	15583256	15565133	C	T	intron_variant	0.000	0.000	0.000	0.000	0.000	0.000	0.000	0.023	0.000
5	rs714205	15583904	15565781	C	G	intron_variant	0.272	0.231	0.125	0.500	0.600	0.472	0.533	0.500	0.269
6	rs750721934	15584822	15566699	T	C	intron_variant	0.000	0.000	0.000	0.000	0.000	0.028	0.000	0.000	0.000
7	rs747549655	15585481	15567358	G	T	intron_variant	0.015	0.000	0.000	0.000	0.000	0.000	0.000	0.019	0.000
8	rs180864908	15590061	15571938	A	G	intron_variant	0.000	0.000	0.000	0.000	0.000	0.000	0.000	0.097	0.077
9	rs768948617	15590562	15572439	A	T	intron_variant	0.010	0.000	0.025	0.000	0.000	0.000	0.000	0.000	0.000
10	NA	15590938	15572815	G	A	intron_variant	0.010	0.000	0.000	0.000	0.000	0.000	0.000	0.000	0.000
11	rs1315695859	15591082	15572959	G	A	intron_variant	0.000	0.000	0.000	0.000	0.020	0.000	0.000	0.000	0.000
12	NA	15591377	15573254	T	C	intron_variant	0.000	0.000	0.000	0.000	0.000	0.000	0.044	0.000	0.000
13	rs202137736	15591485	15573362	T	C	intron_variant	0.000	0.000	0.000	0.000	0.000	0.000	0.056	0.000	0.000
14	NA	15592980	15574857	T	C	intron_variant	0.000	0.000	0.000	0.000	0.000	0.000	0.000	0.005	0.000
15	NA	15596108	15577985	T	C	intron_variant	0.010	0.013	0.000	0.000	0.020	0.000	0.000	0.000	0.000
16	rs753061882	15596934	15578811	G	A	intron_variant	0.000	0.000	0.000	0.000	0.000	0.000	0.000	0.005	0.000
17	rs183880501	15597738	15579615	A	C	intron_variant	0.000	0.026	0.000	0.000	0.000	0.000	0.000	0.000	0.000
18	rs4646150	15599581	15581458	C	T	intron_variant	0.000	0.000	0.000	0.000	0.000	0.000	0.000	0.009	0.077
19	rs4646188	15601343	15583220	A	G	intron_variant	0.154	0.167	0.000	0.042	0.000	0.000	0.000	0.028	0.000
20	NA	15606324	15588201	T	C	intron_variant	0.000	0.000	0.000	0.000	0.000	0.000	0.044	0.000	0.000
21	NA	15607032	15588909	C	T	intron_variant	0.000	0.000	0.000	0.000	0.000	0.000	0.044	0.000	0.000
22	rs1262491345	15608365	15590242	T	A	intron_variant	0.000	0.000	0.000	0.000	0.000	0.000	0.000	0.009	0.000
23	rs1243923033	15610187	15592064	T	G	intron_variant	0.010	0.000	0.000	0.000	0.000	0.000	0.000	0.000	0.000
24	rs2285666	15610348	15592225	C	T	intron_variant	0.277	0.282	0.125	0.500	0.600	0.472	0.511	0.495	0.269
25	rs936854189	15613718	15595595	C	T	intron_variant	0.000	0.000	0.000	0.000	0.000	0.000	0.000	0.005	0.000
26	rs6632677	15614872	15596749	G	C	intron_variant	0.000	0.000	0.000	0.000	0.000	0.083	0.056	0.111	0.000
27	rs767609706	15615697	15597574	A	T	intron_variant	0.000	0.000	0.000	0.000	0.000	0.000	0.011	0.000	0.000
28	rs532429719	15616496	15598373	G	A	intron_variant	0.000	0.000	0.000	0.000	0.080	0.000	0.000	0.000	0.000
29	rs1978124	15618063	15599940	T	C	intron_variant	0.505	0.526	0.175	0.604	0.880	0.944	0.911	0.903	0.846
30	rs188512350	15618337	15600214	G	A	intron_variant	0.015	0.039	0.000	0.000	0.000	0.000	0.000	0.000	0.000
31	rs914799762	15618360	15600237	G	A	intron_variant	0.000	0.000	0.000	0.000	0.000	0.028	0.000	0.000	0.000
32	rs767198373	15618432	15600309	C	G	intron_variant	0.000	0.000	0.000	0.000	0.000	0.028	0.000	0.000	0.000
33	rs368655410	15618933	15600810	G	A	coding_sequence_variant, synonymous_variant	0.015	0.000	0.000	0.000	0.000	0.000	0.000	0.019	0.000
34	NA	15619646	15601523	T	C	intron_variant	0.000	0.000	0.000	0.000	0.000	0.000	0.000	0.019	0.000
35	rs186914723	15619670	15601547	G	A	ntron_variant, genic_upstream_transcript_varian	0.020	0.000	0.000	0.000	0.000	0.000	0.000	0.000	0.000
36	rs190509934	15620340	15602217	T	C	upstream_transcript_variant	0.005	0.000	0.000	0.000	0.120	0.000	0.000	0.000	0.000

SEA_M Southeast Asia Mainland

SEA_I Southeast Asia Island
