# Supplementary material for: Genetic Association of ACE2 rs2285666 Polymorphism With COVID-19 Spatial Distribution in India
Source: Front Genet. 2020 Sep 25;11:564741. doi: 10.3389/fgene.2020.564741 (PMC7545580; doi:10.3389/fgene.2020.564741)
Supplement: Supplementary Table 2 — The summary of data used in present analyses. [file Data_Sheet_2.PDF]

Region	Population Groups	Allele Frequency (T)	Freq. of cases (May 2020)	Freq. of cases (August 2020)	CFR (August 2020)	References
Andhra Pradesh	Telugu	0.546	0.038	0.112	0.009	1000 genome and present study
Arunanchal Pradesh	Nyshi_Bom_Mro_Oraon	1.000	0.000	0.001	0.002	present study
Assam	Assam_Mix	0.510	0.002	0.030	0.003	present study
Bihar	Bihar_Mix	0.575	0.007	0.040	0.005	present study
Gujarat	Gujarati	0.400	0.112	0.029	0.034	1000 genome
Haryana	Ror	0.550	0.012	0.017	0.011	Pathak et al. 2018
Jharkhand	Santhal_Munda_Oraon	0.700	0.002	0.009	0.011	present study
Jammu & Kashmir	Brahmins	0.443	0.019	0.011	0.019	present study
Kerala	Cochin_Jews and Mix	0.875	0.020	0.018	0.004	present study
Meghalaya	Garo and Khasi	0.756	0.001	0.001	0.003	Tatte et al. 2019 and present study
Rajasthan	Gujjar	0.586	0.090	0.023	0.014	Pathak et al. 2018
Tamilnadu	Tamil and Sri Lankan Tami	0.490	0.076	0.124	0.017	1000 genome and present study
Tripura	Tripuri	1.000	0.000	0.003	0.008	present study
Uttar Pradesh	Brahmins and Kshatriya	0.460	0.068	0.059	0.016	present study
West Bengal	Bengali	0.554	0.021	0.044	0.02	present study
Maharashtra	Marathi_Parsi_Jews	0.330	0.263	0.224	0.033	Chaubey et al. 2017 and present study
Bangladesh	Bangladeshi	0.344	-	-	-	present study
Bangladesh	Khumi	0.500	-	-	-	present study
Bangladesh	Marma	0.750	-	-	-	present study
Bangladesh	Tanchagya	0.500	-	-	-	present study
